# Review of the existing evidence for the relationship between person-organization fit and mental health outcomes in the workplace

**DOI:** 10.13075/ijomeh.1896.02913

**Published:** 2026

**Authors:** Kaja Staszewska, Dorota Merecz-Kot

**Affiliations:** 1 Nofer Institute of Occupational Medicine, Department of Health and Work Psychology, Łódź, Poland; 2 University of Lodz, Department of Psychology of Work, Organisation and Career Counselling, Łódź, Poland

**Keywords:** depression, anxiety, stress, well-being, mental health, P-O fit

## Abstract

Person-organization fit (P-O fit) refers to how well employees feel they match their organization in terms of values, goals, needs, and personal characteristics. Although previous research has primarily focused on the relationship between P-O fit and performance measures, increasing attention has recently been devoted to its potential role in employee mental health. The aim of this review was to summarize the current evidence regarding the relationship between P-O fit and mental health outcomes in occupational settings. The review was conducted in accordance with Preferred Reporting Items for Systematic Reviews and Meta-Analyses 2020 (PRISMA 2020) guidelines. Electronic searches were performed in the PubMed, Scopus, and EBSCO databases for studies published in 2016–2026. Studies were included if they examined the relationship between P-O fit and mental health outcomes among working populations. After the screening and eligibility assessment process, 16 empirical studies were included in the review. The reviewed studies included predominantly cross-sectional designs, although 2 longitudinal studies were also identified. Most studies showed that higher levels of P-O fit were associated with lower levels of stress, burnout, depressive and anxiety symptoms, and better well-being outcomes. Similar associations were reported in different occupational groups and countries. In conclusion, the findings suggest that congruence between employees and their organizational environments may play an important role in employee mental health. However, this review has several limitations, including the predominance of cross-sectional studies. Further longitudinal research is needed to clarify the relationship between P-O fit and employee mental health.

## Highlights

- Person-organization fit was negatively associated with anxiety and depressive symptoms.
- Person-organization fit was negatively associated with stress and burnout.
- Similar associations were observed across occupations and countries.

## INTRODUCTION

The paradigm of person-organization fit (P-O fit) has been the subject of extensive analysis and research for several decades. The P-O fit is commonly conceptualized as the compatibility between individuals and organizations [1]. Muchinsky and Monahan [2] proposed one of the most influential conceptualizations of this construct by distinguishing between supplementary fit and complementary fit. Supplementary fit refers to the similarity between employees and organizations in terms of values, goals, and characteristics, whereas complementary fit refers to the extent to which employees and organizations complement one another by providing characteristics or resources that are lacking in the other [2,3]. Another important perspective on P-O fit is based on the distinction between needs-supplies fit and demands-abilities fit. From the needs-supplies perspective, P-O fit occurs when the organization fulfills employees' needs, preferences, or expectations, whereas the demands-abilities perspective emphasizes the extent to which employees possess the abilities and competencies required to meet organizational demands [4,5]. Importantly, P-O fit should not be treated as an entirely static characteristic. Recent theoretical and review-based work emphasizes that employees may actively manage their fit and misfit experiences, while organizations may use purposeful practices, such as socialization and career-related support, to foster employees' fit over time [6]. Similarly, Sekiguchi [7] suggests that organizations may promote different forms of person-environment fit through human resource practices, including recruitment, selection, orientation, training, socialization, job assignment, internal staffing, and career development. Taken together, these arguments suggest that P-O fit may be, at least to some extent, modifiable at both the individual and organizational levels. However, empirical evidence remains limited, particularly from intervention studies examining whether deliberate efforts to increase P-O fit lead to subsequent improvements in mental health outcomes. In the mainstream of research on P-O fit, attention has been focused primarily on its associations with job performance and job satisfaction [8]. Research findings indicate that P-O fit is associated with employees' job satisfaction, sense of meaning at work, and work engagement [9,10]. Meta-analytic findings have additionally demonstrated that higher levels of P-O fit are related to stronger organizational commitment and lower turnover intentions [10]. Similar associations were also observed in a broader meta-analysis examining multiple forms of workplace fit, including person-organization fit [11]. Longitudinal findings by Chatman [12] further indicated that employees whose values were more congruent with organizational values reported greater job satisfaction and were more likely to remain within the organization over time. Conversely, misfit has been linked to negative consequences for the employee, and, in the longer term, a decline in the quality of occupational functioning [13]. Misfit also has significant consequences for the organization, as it may lead to sickness absence, employee turnover, and the necessity of incurring additional costs associated with the recruitment and training of new staff. Mental health problems among employees are increasingly recognized as a major public health and organizational challenge, contributing to productivity loss, sickness absence, and impaired occupational functioning [14,15]. Chronic exposure to occupational stressors has been associated with burnout, depressive symptoms, anxiety, emotional exhaustion, and impaired psychological well-being [16,17]. Due to increasing concerns about employee mental health, it is important to determine whether P-O fit may be associated with employee well-being. However, evidence regarding the relationship between P-O fit and mental health remains fragmented, and to the authors' knowledge, no previous review has specifically synthesized findings concerning the relationship between P-O fit and employee mental health outcomes.

The aim of this review was to summarize the current state of knowledge regarding the relationship between P-O fit and mental health indicators, including well-being, stress, burnout, depression, and anxiety.

## METHODS

### Search strategy

This review was conducted in accordance with the Preferred Reporting Items for Systematic Reviews and Meta-Analyses 2020 (PRISMA 2020) guidelines [18]. To identify relevant studies, the following electronic bibliographic databases were searched: EBSCO, PubMed, and Scopus. The search was conducted in April 2026. The review was limited to studies published between 2016 and 2026.

The search strategy was based on 2 groups of keywords. In the first stage, terms related to P-O fit were used (“person-organization fit” OR “P-O fit” OR “person-organisation fit”). In the second stage, terms related to mental health (“mental health” OR “psychological health”) and mental health outcomes were used, including well-being (“wellbeing” OR “well-being” OR “psychological well-being”), stress (“stress” OR “job stress” OR “occupational stress” OR “work stress” OR “distress”), burnout (“burnout” OR “exhaustion” OR “emotional exhaustion”), as well as depression and anxiety. The keywords were combined using Boolean operators and adapted to the requirements of each database.

### Inclusion and exclusion criteria

Studies were included if:

– they were published in peer-reviewed journals between 2016 and 2026;
– they were written in English or Polish;
– they included working populations, regardless of occupation, industry, or country;
– they included analyses of the relationship between P-O fit and mental health outcomes, such as well-being, stress, burnout, depression, or anxiety;
– they used standardized measures;
– they were available in full-text open-access form.

Review articles, systematic reviews, meta-analyses, theoretical papers, case studies, and studies involving students or volunteers were not considered. The review also omitted studies focused on the development, adaptation, or validation of measures and studies examining person-organization fit solely as a moderator.

The retrieved records were evaluated using a 2-stage process. In the first stage, after removing duplicates, the titles and abstracts of the identified studies were screened independently by 2 authors. In the second stage, the full texts of potentially relevant articles were reviewed, and only studies meeting the inclusion criteria were retained. Disagreements were resolved through discussion and consensus between the 2 authors. The following data were extracted from the included studies: first author's name, country, study design, participant characteristics, exposure variables, mental health outcomes, measurement tools, main findings, and study limitations.

### Quality assessment

The methodological quality of the included studies was assessed using Joanna Briggs Institute (JBI) critical appraisal tools appropriate for the study design. Cross-sectional studies were evaluated using the *JBI Critical Appraisal Checklist for Analytical Cross-Sectional Studies*, while longitudinal/cohort studies were assessed using the *JBI Critical Appraisal Checklist for Cohort Studies* [19]. The selected appraisal tools evaluated several methodological aspects, including clearly defined inclusion criteria, detailed description of study subjects and settings, validity and reliability of exposure measurement, use of objective and standard criteria for measurement of the condition, identification of confounding factors, strategies to deal with confounding factors, validity and reliability of outcome measurement, completeness of follow-up and strategies to address incomplete follow-up in cohort studies, and appropriateness of statistical analyses. The quality assessment was conducted independently by 2 authors.

## RESULTS

### Study selection and characteristics

Figure 1 presents the study selection process for review. A total of 239 records were identified through database searching, including Scopus (N = 108), EBSCO (N = 102), and PubMed (N = 29). After removing duplicates, 138 records remained for title and abstract screening. Following the screening process, 45 full-text articles were assessed for eligibility. Ultimately, 16 studies were included in the systematic review. Table 1 shows the included studies. Burnout was the most frequently assessed outcome (N = 7), followed by stress (N = 5), well-being (N = 2), and depression and anxiety (N = 2). Most studies employed a cross-sectional design (N = 14), whereas 2 studies used longitudinal designs: one was a 2-wave longitudinal study with time-lagged measurement, and one was a 3-wave longitudinal panel study. The included studies were conducted in diverse occupational and cultural contexts, including samples from Europe, Asia, North America, and Africa. The occupational groups included, among others, healthcare workers, nurses, teachers, academic staff, office-based employees, service-sector employees, IT employees, and industrial workers. Person-organization fit was assessed using various standardized self-report measures. These included direct measures of overall perceived P-O fit, as well as measures assessing specific dimensions of fit, including supplementary fit, needs-supplies fit, and demands-abilities fit. Measures developed by Cable and Judge [36] and Cable and DeRue [37], as well as their adaptations or modifications, were frequently used. Some studies used broader person-environment fit measures that included a P-O fit subscale, while 1 study reported results in terms of person-organization misfit. Thus, although all included studies assessed compatibility between employees and organizations, the operationalization of P-O fit varied across studies.

**Figure 1.**
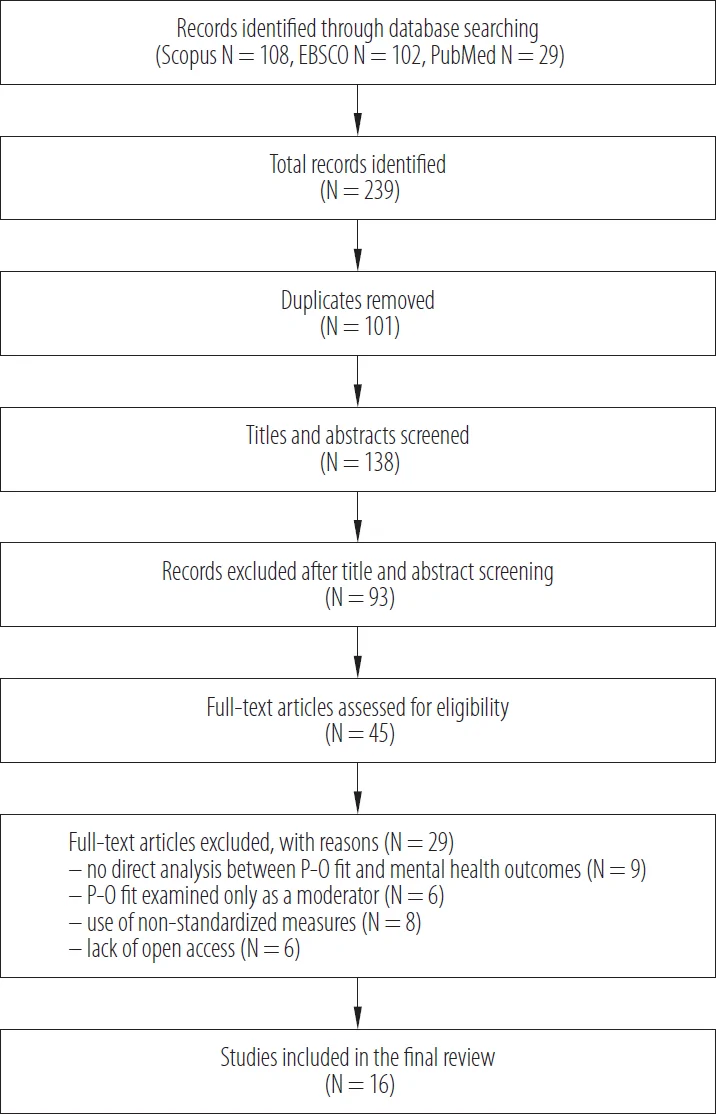
PRISMA flow diagram of the selection process in the study on the relationship between person-organization fit (P-O fit) and mental health outcomes in occupational settings, 2016–2026, Europe, Asia, North America, and Africa

**Table 1.** Description of studies that examined the relationship between person-organization fit (P-O fit) and mental health outcomes, 2016–2026, Europe, Asia, North America, and Africa

Study	Country	Study design	Participants	Exposure variable	Mental health outcomes	Measurement tools	Main findings	Limitations reported in the original studies
Kilroy et al., 2017 [20]	Canada	2-wave longitudinal study with time-lagged measurement	185 health care employees; 91% women; age M = 49 years	P-O fit	burnout	*Perceived Person-Organization Fit Scale* (Cable and Judge, 1996 [36]; 3 items); *Maslach Burnout Inventory – Human Services Survey* (Maslach et al., 1996; 10 items)	P-O fit was negatively associated with emotional exhaustion (β = –0.331, p < 0.001) and depersonalization (β = –0.363, p < 0.001)	single-organization sample (Canadian hospital); time-lagged design with only 2 waves of data collection; possible influence of contextual factors not examined
Waszkowska et al., 2017 [21]	Poland	cross-sectional study	715 middle-level managers; 381 women, 334 men; age M = 42.7 years	supplementary P-O fit	stress	*Person-Organization Fit Questionnaire* (supplementary P-O fit subscale; Czarnota-Bojarska, 2010; 16 items); PSS-10 (Cohen et al., 1983; 10 items)	P-O fit was negatively associated with perceived stress (β = –0.42, p < 0.001) and this relationship was partially mediated by effort-reward imbalance	exploratory, cross-sectional design (limits causal inference); use of general stress measure (PSS-10, not specific to occupational stress); limited generalizability (sample restricted to middle-level managers)
Andela and van der Doef, 2019 [22]	France	cross-sectional study	571 employees; service sector; 326 women, 245 men; age M = 37 years	person-environment fit (P-J fit, P-O fit, P-G fit, P-S fit)	burnout	*Perceived Person-Environment Fit Scale* (Person-Organization Fit subscale) (Chuang et al., 2016); *Maslach Burnout Inventory – General Survey* (Schaufeli et al. 1996; 16 items)	P-O fit was negatively associated with emotional exhaustion (β = –0.29, p < 0.01) and cynicism (β = –0.31, p < 0.01), and was not significantly associated with professional efficacy (β = 0.06, p > 0.05)	cross-sectional design; limited control of background variables (potential confounders); reliance on self-report measures (common method bias)
Akkaya et al., 2020 [23]	Turkey	cross-sectional study	393 academics working at state and foundation universities; 244 women, 149 men; age not reported	P-O fit	burnout	*Person-Organization Fit Scale* (Cable and Judge, 1997; adapted by Ünal and Turgut, 2013; 40 items); *Maslach Burnout Scale* (Maslach and Jackson, 1981; adapted by Ergin, 1993; 22 items)	higher levels of person-organization misfit were associated with greater emotional exhaustion (r = 0.314, p < 0.01), depersonalization (r = 0.168, p < 0.01), and total burnout (r = 0.284, p < 0.01)	cross-sectional design; sample limited to academics from universities in Istanbul
Padmasiri and Kailasapathy, 2020 [24]	Sri Lanka	cross-sectional study	393 employees; operational level workers in apparel industry; 51% male; age distribution: 8.6% <20 years, 56.8% 20–30 years, 21% 30–40 years, 13.6% >40 years	person-environment fit (P-J fit, P-G fit, P-S fit, P-O fit, and person-family fit)	psychological well-being	*Perceived Person-Environment Fit Scale* (Chuang et al., 2016; P-O scale: 7 items); *Psychological Well-Being Scale* (Bradburn, 1969; 10 items)	P-O fit was not significantly associated with psychological well-being (p > 0.05)	cross-sectional design (no causal inference); convenience sampling; limited generalizability
Johan and Yusuf, 2022 [25]	Indonesia	cross-sectional study	88 employees; leasing industry; 78% men, 22% women; age not reported	P-O fit	job stress	*Person-Organization Fit Scale* (Cable and DeRue, 2002 [37]; 9 items); *Job Stress Scale* (adapted from Crank et al., 1995; 6 items)	P-O fit was negatively associated with job stress (β = –0.30, p < 0.05)	small sample size (low generalizability); limited generalizability to other countries and contexts; need for larger and cross-cultural samples in future research; focus on a limited set of variables (need to include additional predictors, such as other determinants of counterproductive work behavior)
Yang and Yasmin, 2022 [26]	China	cross-sectional study	296 employees; education sector; 71% male; majority <36 years	high-performance HR practices, P-O fit, P-J fit	job stress	*Person-Organization Fit Scale* (O'Reilly and Chatman, 1986; 4 items); *Job Stress Scale* (Motowidlo et al., 1986; 4 items)	P-O fit was negatively associated with job stress (β = –0.80, 95% CI: –1.07–0.53) and mediated the relationship between high-performance HR practices and job stress (β = –0.57, 95% CI: –0.95–(–0.18))	cross-sectional design (limits causal inference); self-report data (common method bias); limited set of HR practices included; convenience sampling (limited generalizability)
Zang and Chen, 2022 [27]	China	cross-sectional study	637 kindergarten teachers; 98% female; 2% male	P-O fit, job satisfaction	burnout	*Person-Organization Fit Scale* (Wang Ling, adapted from Cable and DeRue, 2002 [37]; 9 items); *Maslach Burnout Inventory – Educators Survey* (22 items)	P-O fit was negatively correlated with teacher burnout (r = –0.510, p < 0.001). Demand-supply fit showed the strongest negative correlation with burnout (r = –0.554, p < 0.001).	sample limited to kindergarten teachers from Henan Province, China; cross-sectional design limiting causal inference; other organizational and mediating factors were not examined
Ali et al., 2023 [28]	Egypt	cross-sectional study	373 frontline workers and supervisors from the soap and detergents industry; mean age not reported; gender distribution not reported; additional qualitative sample: 14 production-line and QC supervisors	P-O fit (supplementary fit, complementary fit – demand-abilities fit, need-supplies fit)	MHIW (anxiety and depression, psychosocial dysfunction, loss of confidence)	*Perceived Person-Organization Fit Scale* (Cable and Judge, 1996 [36]; 9 items); *General Health Questionnaire* (GHQ-12; Goldberg, 1988; 12 items)	need-supplies fit (β = –0.53, p < 0.05) and supplementary fit (β = –0.43, p < 0.05) were negatively associated with MHIW; MHIW mediated the relationship between P-O fit dimensions and workers' productivity growth during COVID-19	single-country and single-industry sample (Egyptian soap and detergent industry); focus on frontline routine workers only
Jung et al., 2024 [29]	Germany	cross-sectional study	68 employed adults; 57% male, 43% female; age M = 50.5 years	person-environment fit (P-O fit, demands-abilities fit, needs-supplies fit)	depression, anxiety	*Perceived Person-Environment Fit Scale* (*Person-Organization Fit Scale*; Cable and DeRue, 2002 [37]; 3 items); *Center for Epidemiologic Studies Depression Scale* (Radloff, 1977; 20 items); *Generalized Anxiety Disorder Scale* (GAD-7) (Spitzer et al., 2006; Löwe, 2008; 7 items)	symptoms of depression were negatively associated with P-O fit (β = –0.185, p < 0.001), demands-abilities fit (β = –0.307, p < 0.001), and needs-supplies fit (β = –0.247, p < 0.001); symptoms of anxiety were also negatively associated with P-O fit (β = –0.135, p = 0.019), demands-abilities fit (β = –0.207, p = 0.006), and needs-supplies fit (β = –0.189, p = 0.003)	subjective (self-report) assessment of fit; cross-sectional design (limits causal inference and directionality); lack of identification of risk groups based on symptom severity and person-environment fit; no differentiation between occupational sectors
Özlü and Türe, 2024 [30]	Turkey	cross-sectional study	500 nurses working in a university hospital; 74.6% women; age M = 31.27 years	P-O fit, perceived organizational support	job stress	*Person-Organization Fit Scale* (Netemeyer et al., 1997; Turkish adaptation by Turunç and Çelik, 2012; 4 items); *Perceived Job Stress Scale* (Cohen and Williamson, 1988; Turkish adaptation by Baltaş, 1988)	P-O fit did not have a statistically significant effect on perceived job stress (β = 0.119, p > 0.05)	limited generalizability (nurses from 1 university hospital); interview-based data collection (participants may not always express their thoughts accurately and clearly)
Zeng and Hu, 2024 [31]	China	cross-sectional study	477 employees from the IT sector; 53.7% male, 46.3% female; age M = 33.71 years	P-O fit, P-J fit, work pressure	burnout	*Person-Organization Fit Scale* (Cable and Judge, 1996 [36]; 7 items); *Burnout Scal*e (Li and Shi, 2003; 5 items)	P-O fit was not significantly associated with job burnout (B = −0.044, p > 0.05), but was negatively associated with work pressure (B = −0.276, p < 0.01); work pressure was positively associated with job burnout (B = 0.761, p < 0.01)	cross-sectional design (limits causal inference); limited generalizability (IT employees from one region in China); focus on a single mediator (work pressure); lack of consideration of additional mediators and moderators (e.g., personality traits)
Cavicchioli et al., 2025 [32]	Italy	cross-sectional study	144 employees from an AI company; 75.95% men; age M = 34 years	P-O fit, work-life enrichment, work-life conflict, work engagement, job satisfaction, off-hours digital actions, weekly virtual meetings	work-related stress	*Person-Organization Fit Scale* (Ng and Feldman, 2009; 3 items); *Work-related Stress Scale* (adapted from Goldberg and Williams, 1988; 3 items)	P-O fit significantly reduced work-related stress (F = 23.217, p < 0.01)	short period of Microsoft 365 click metadata collection; small sample size; single-organization sample; predominantly male sample; self-report measures
Paliga et al., 2025 [33]	Poland	cross-sectional study	804 employees performing office-based jobs (on-site, hybrid, and remote workers); 71% women, 28% men, and 1% identifying as other or providing no response; age M = 31.88 years	P-O fit, job performance, work modality	positive and negative job-related affective well-being	*The Person-Organization Fit Questionnaire* (Czarnota-Bojarska, 2012; 34 items); *Job-related Affective Well-Being Scale* (Van Katwyk et al., 2011; Polish adaptation by Basińska et al., 2014; 12 items)	P-O fit had a direct positive effect on positive job-related affective well-being (B = 0.69, 95% CI: 0.61–0.78) and a direct negative effect on negative job-related affective well-being (B = –0.71, 95% CI: –0.79–(–0.63))	cross-sectional design; self-report measures; predominantly female sample; other predictors of job performance require further analysis
Flatau-Harrison et al., 2025 [34]	Belgium	3-wave longitudinal panel study	193 employees at baseline; final longitudinal sample N = 147; 86 women; 76.2% full-time employees; employees from profit, non-profit, and government sectors; 39.5% executive/administrative roles, 35.4% professional employees, 25.2% management positions	P-O fit, organizational trust, job crafting toward strengths, job crafting toward interests	burnout	*Person-Organization Fit Scale* (Cable and DeRue, 2002 [37]; 3 items); *Utrecht Burnout Scale* (Schaufeli and Van Dierendonck, 2000; 9 items)	P-O fit negatively predicted exhaustion (B = –0.18, p < 0.05) and cynicism (B = –0.20, p < 0.05); the indirect effect of P-O fit on exhaustion through organizational trust was significant (indirect effect = –0.09, 95% CI: –0.16–(–0.02)), indicating partial mediation	3-wave design may not fully rule out reverse causality; future studies should use more comprehensive longitudinal models to distinguish between-person and within-person variation; reliance on self-report measures
Zhou et al., 2025 [35]	China	cross-sectional study	588 teaching staff members from vocational colleges; 67% women, 33% men	P-O fit, work overload	burnout	*Person-Organization Fit Scale* (Cable and DeRue, 2002 [37]; 6 items); *Maslach Burnout Inventory* – *General Survey*	P-O fit negatively predicted job burnout (β = –0.413, t = 10.068, p < 0.001); job burnout mediated the relationship between P-O fit and quiet quitting (β = –0.191, t = 5.177, p < 0.001)	sample limited to vocational colleges in China; cross-sectional design limiting causal inference; other potential moderators (e.g., perceived organizational support, psychological resilience, professional identity) were not examined

HR – human resources; MHIW – mental health issues at workplace; P-G fit – person-group fit; P-J fit – person-job fit; P-S fit – person-supervisor fit; PSS-10 – *Perceived Stress Scale*.

β – standardized regression coefficient; B – unstandardized regression coefficient.

### Well-being outcomes

The available findings regarding the relationship between P-O fit and well-being were mixed. Both studies used across-sectional design. Paliga et al. [33] examined 804 office-based employees working in on-site, hybrid, and remote arrangements and demonstrated that P-O fit had a direct positive effect on positive job-related affective well-being and a direct negative effect on negative job-related affective well-being. The observed relationships were consistent across all examined work modalities. In contrast, Padmasiri and Kailasapathy [24] studied 393 operational-level employees in the apparel industry and did not identify a statistically significant relationship between P-O fit and psychological well-being. However, the broader construct of person-environment fit, which included person-job fit, person-group fit, person-supervisor fit, and person-family fit, was positively associated with psychological well-being.

### Work-related stress

Five studies examined the relationship between P-O fit and work-related stress. Most used a cross-sectional design and generally showed that employees reporting higher levels of P-O fit tended to experience lower stress levels. Waszkowska et al. [21], in a study of 715 middle-level managers employed in large enterprises, found that supplementary fit was negatively associated with perceived stress. Managers whose values, goals, and expectations were less congruent with those of the organization reported higher stress levels. The authors additionally noted that the protective role of P-O fit may be reduced under conditions of effort-reward imbalance. Negative associations between P-O fit and job stress were also reported in studies conducted among employees in the Indonesian leasing industry and among employees in the Chinese education sector [25,26]. Similar findings were observed by Cavicchioli et al. [32], who reported lower levels of work-related stress among employees perceiving higher P-O fit in highly digitalized work environments. In contrast, Özlü and Türe [30], in a study among nurses employed at a university hospital, did not identify a significant relationship between P-O fit and job stress. In their study, P-O fit also did not mediate the relationship between perceived organizational support and stress.

### Depressive and anxiety symptoms

Research addressing depressive and anxiety symptoms consistently indicated that better fit between employees and their work environment was associated with lower symptom severity. Jung et al. [29], based on a population-based sample of 568 employed adults, found negative associations between symptoms of depression and anxiety and all examined dimensions of fit, including P-O fit, demands-abilities fit, and needs-supplies fit. Employees reporting greater congruence with their work environment tended to report lower severity of depressive and anxiety symptoms, with the strongest associations observed for demands-abilities fit. Comparable findings were reported by Ali et al. [28] among 373 frontline workers and supervisors from the Egyptian soap and detergent industry during the COVID-19 pandemic. The authors demonstrated that supplementary fit and needs-supplies fit were negatively associated with workplace mental health issues, including anxiety and depression, psychosocial dysfunction, and loss of confidence. In addition, workplace mental health issues mediated the relationship between fit and workers' productivity during the pandemic.

### Burnout symptoms

Most studies examining burnout symptoms used cross-sectional designs, although one 2-wave longitudinal study with time-lagged measurement and one 3-wave longitudinal panel study were also identified. The studies were conducted in diverse occupational groups, including kindergarten teachers (N = 637), service-sector employees (N = 571), and teaching staff members (N = 588) [22,27,35]. Taken together, the available findings generally indicated that higher levels of P-O fit were associated with lower levels of burnout symptoms, particularly emotional exhaustion and depersonalization/cynicism [22]. Similar findings were also observed in longitudinal studies. Kilroy et al. [20], in a 2-wave longitudinal study among Canadian health care employees, demonstrated that P-O fit was negatively associated with emotional exhaustion and depersonalization. Likewise, Flatau-Harrison et al. [34], using a 3-wave longitudinal panel design, found that P-O fit negatively predicted exhaustion and cynicism. Zeng and Hu [31], based on a study of 477 employees from the Chinese IT sector, did not identify a significant direct relationship between P-O fit and burnout. However, P-O fit was negatively associated with work pressure, while work pressure was positively associated with burnout symptoms, suggesting that work pressure may represent an important mechanism linking fit and burnout. In contrast, Akkaya and Serin [23] reported that higher levels of person-organization misfit were associated with greater emotional exhaustion, depersonalization, and overall burnout among Turkish academics. Importantly, in this study P-O fit was operationalized using a deviation score calculated from discrepancies between employees' ratings of ideal and actual organizational conditions, meaning that higher scores indicated greater misfit rather than higher levels of fit.

### Summary of methodological quality and limitations

The methodological quality of the included studies was generally acceptable. All included studies used standardized measures to assess P-O fit and mental health outcomes, as this was one of the inclusion criteria of the review. The final column of Table 1 summarizes the key limitations reported by the authors of the original studies and does not represent a separate numerical quality score or quality category. Several methodological limitations should also be noted. Most studies used cross-sectional designs, which makes it difficult to draw causal conclusions. Many studies were based solely on self-report measures, which may have increased the risk of common method bias. In some studies, information regarding participant characteristics, such as age, was limited or incomplete. Information about potential confounding factors and their control was also not always fully reported.

### Discussion

The findings of the present review are generally consistent with the theoretical assumptions of P-O fit, which posit that compatibility between employees and their organizations is associated with more favorable individual outcomes [1]. The reviewed studies largely support this assumption, as higher levels of P-O fit were generally associated with better mental health and well-being among employees. Across the reviewed studies, higher levels of fit were generally associated with lower levels of burnout, stress, depressive and anxiety symptoms, as well as more favorable well-being outcomes. Similar patterns were observed across different occupational groups and cultural contexts, suggesting that the relationship between P-O fit and employee's mental health may not be limited to a specific profession, industry, or organizational setting. In particular, the findings indicated relationships between P-O fit and lower levels of emotional exhaustion and depersonalization/cynicism [20,22,34]. Although the direction of the observed associations was largely consistent across studies, their magnitude varied from relatively weak to strong depending on the mental health outcome, occupational group, and organizational context. However, due to the predominance of cross-sectional studies, the causal direction of these relationships remains unclear. Therefore, future longitudinal research is needed to determine whether congruence between employees and their organizational environments may represent a protective factor against the negative effects of psychosocial hazards in the work environment. Several studies additionally indicated that the relationship between P-O fit and mental health outcomes may be partially explained by mediating mechanisms such as work pressure and effort–reward imbalance, while one study suggested that perceived organizational support may represent an important predictive factor associated with both higher levels of P-O fit and lower occupational stress [21,30,31]. These findings are consistent with previous meta-analytic and review evidence demonstrating that adverse psychosocial working conditions contribute to poorer mental health outcomes among employees [15,16]. Similar conclusions were reported by Merecz and Andysz [13], who indicated that obtaining an optimal level of P-O fit may be beneficial not only for organizations, through lower turnover, better work effectiveness and commitment, but also for employees' health. Importantly, previous reviews and meta-analyses have primarily focused on the associations between P-O fit and work attitudes, job satisfaction, organizational commitment, and turnover intentions [10,11]. Given the growing number of work-related mental health problems, it is increasingly important to better understand how P-O fit may be linked to employee well-being. The findings of this review may also have important practical implications. Higher levels of congruence between employees and organizations in terms of values, goals, and needs may reduce occupational stress and burnout and support employees' psychological well-being. Furthermore, contemporary workplaces are undergoing substantial changes associated with digitalization, evolving organizational structures, flexible forms of employment, and changing employee expectations and values. These transformations may also influence how P-O fit is experienced and understood in modern work environments. Future research could focus on how changes in work environments and organizational demands may influence the relationship between P-O fit and employee mental health.

### Limitations and future directions

Despite the interesting results highlighting the importance of P-O fit for mental health, the present review has several limitations. First, the search strategy and eligibility criteria may have resulted in the exclusion of some relevant studies. Only studies published in English or Polish and available in full-text open-access form were included, which may have excluded relevant articles published in specialist or high-impact journals with restricted access. Second, although the broad international scope of the included studies is a strength of this review, cultural and organizational differences across countries should be considered when interpreting the findings. Differences in organizational values, hierarchy, and employee-organization relationships may limit the direct comparability of results obtained in different cultural contexts. Third, most studies used cross-sectional designs and self-report measures, which limit the possibility of drawing causal conclusions and may increase the risk of common method bias. An additional limitation was the heterogeneity in the measurement of P-O fit and the reporting of results across the included studies. As a result, it was not possible to directly compare the strength of associations between supplementary and complementary fit and mental health outcomes.

Future research should include more longitudinal studies examining how P-O fit may influence employee mental health. This appears particularly important given that most of the available evidence is based on cross-sectional studies, which limits the possibility of drawing causal conclusions regarding the relationship between P-O fit and mental health outcomes. Additional studies are also needed to better understand which dimensions of P-O fit may be most strongly associated with different mental health outcomes across occupational and cultural contexts. Future research could also focus on organizational interventions aimed at improving P-O fit and examine whether such interventions may contribute to reduced occupational stress, lower burnout levels, and better psychological well-being among employees.

## CONCLUSIONS

This review indicates that higher levels of P-O fit are generally associated with more favorable mental health outcomes among employees, including lower levels of burnout, stress, depressive and anxiety symptoms, and better well-being. However, the predominance of cross-sectional studies limits causal interpretation. Future longitudinal studies could also examine whether and which organizational actions aimed at improving P-O fit may contribute to reduced occupational stress, lower burnout levels, and better psychological well-being among employees.
